# Efficacy and safety of persimmon leaf formulated with green tea and sophora fruit extracts (BLH308) on hair growth: A randomized, double‐blind, placebo‐controlled clinical trial

**DOI:** 10.1111/srt.13448

**Published:** 2023-08-22

**Authors:** Seoyoon Ham, Young In Lee, In Ah Kim, Jangmi Suk, Inhee Jung, Jong‐Moon Jeong, Ju Hee Lee

**Affiliations:** ^1^ Department of Dermatology & Cutaneous Biology Research Institute Yonsei University College of Medicine Seoul Republic of Korea; ^2^ Scar Laser and Plastic Surgery Center Yonsei Cancer Hospital Seoul Republic of Korea; ^3^ Global Medical Research Center Seoul Republic of Korea; ^4^ Department of Bioscience College of Engineering The University of Suwon Hwasung‐si Republic of Korea

**Keywords:** clinical trial, efficacy, green tea, hair growth, persimmon leaf, safety, sophora fruit extract (BLH308)

## Abstract

**Background:**

Recent research suggests that persimmon leaf extract (PLE) has an effect on inflammatory skin diseases. Previously, PLE is revealed to inhibit not only nitric oxide production but also inducible nitric oxide synthase (iNOS) and cyclooxygenase‐2 (COX‐2) expression levels in mouse macrophages in vitro. Moreover, it significantly reduced IL‐6 production and 5α‐reductase expression in human follicle dermal papilla cells (HFDPCs). This study aimed to determine whether the PLE‐containing BLH308 complex improves hair growth in clinical trials.

**Materials and Methods:**

A total of 88 participants were recruited, and were instructed to orally take BLH308 or the placebo twice a day for 24 weeks. The mean age of the test group was 38.52 ± 7.98 years and that of placebo group was 38.98 ± 8.80 years. The study was conducted for 24 weeks, and hair density, thickness, and gloss were evaluated. All participants completed a satisfaction survey questionnaire.

**Results:**

The test group showed significantly increased hair density and hair diameter at week 24 compared with the placebo group (*p* = 0.0015 and *p* = 0.0001, respectively). Although not statistically significant, the degree of gloss also showed higher improvement in the test group compared to the placebo group.

**Conclusions:**

Our data demonstrated that oral consumption of the BLH308 complex containing PLE significantly increased hair density and thickness compared to the placebo group, showing its possible role in promoting hair growth.

## 1 INTRODUCTION

Persimmon (*Diospyros kaki*) leaf extract (PLE) has been widely used in East Asian traditional medicine for its antioxidant properties.^1^ It is mostly composed of anti‐inflammatory flavonoids, such as quercetin, isoquercetin, hyperin, astragalin, and kaempferol.^2^ Recent studies have revealed that the extract from the persimmon leaves possesses a range of biological properties, including radial scavenging, neuroprotection, thrombosis inhibition, anti‐atherosclerosis, and anti‐allergy.^3^ The antioxidant and anti‐inflammatory effects of PLE can also lead to a photoprotective and anti‐aging effect,^4^ which can affect the maintenance of healthy hair growth activity.

A few dermatologic studies have explored the effect of PLE on inflammatory dermatologic diseases. PLE was reported to have an inhibitory effect on T cell activation via reduction of interleukin (IL)‐2 production in vitro; an additional in vivo animal model of atopic dermatitis resulted in reduced IgE levels, mast cell infiltration, and pro‐inflammatory cytokine expressions, especially Th2 cytokines, after oral administration of PLE, suggesting its role in inflammatory skin diseases such as atopic dermatitis.^1^ Another study on the effect of PLE in atopic dermatitis assessed its clinical therapeutic effect in NC/Nga mice, showing that oral administration of PLE for 4 weeks into the atopic dermatitis mouse model with overt dermatitis resulted in dose‐dependent decreases in not only the skin severity score but also in transepidermal water loss (TEWL), serum IgE, and skin scratching behavior.^5^

Although the anti‐inflammatory effect of PLE on inflammatory skin diseases such as atopic dermatitis seems obvious, the effect of PLE on other dermatologic conditions remains unknown. The effect of PLE in vitro was investigated, and it was found that PLE inhibited not only nitric oxide production but also inducible nitric oxide synthase (iNOS) and cyclooxygenase‐2 (COX‐2) expression levels in mouse macrophages. Moreover, it significantly reduced IL‐6 production as well as the expression level of 5α‐reductase in human follicle dermal papilla cells (HFDPCs).^6^ Recently, novel therapeutic interventions for promoting hair growth in alopecia have been garnering considerable scientific interest, such as platelet‐rich plasma^7^ and the use of oral supplements containing hydrolyzed collagen and aminoacids.^8^ Therefore, in this study, we designed a randomized double‐blind placebo‐controlled study to evaluate the efficacy and safety of BLH308, the study product, which contained PLE as the major active ingredient formulated with green tea (*Camellia sinensis*) and sophora fruit (Sophora japonica) on hair growth and overall hair health when orally consumed. A total of 88 healthy Korean adults were recruited and were instructed to orally take BLH308 or a placebo containing PLE twice daily for 24 weeks. Consequently, the participants who consumed the test product showed significantly increased hair density and hair diameter compared with the control group. In addition, although not statistically significant, the degree of gloss assessed by the glossymeter also showed higher improvement in the test group compared to the control group.

## 2 MATERIALS AND METHODS

### 2.1 Study participants

Participants in this study were recruited between the ages of 19 and 60 without any underlying medical conditions (men = 34, women = 54). The mean age of the test group was 38.52 ± 7.98 years, and that of the placebo group was 38.98 ± 8.80 years (Table 1). These participants had no history of hair disorders or alopecia. The exclusion criteria were as follows: (1) underlying dermatologic disorders or other medical conditions undergoing any medical treatment; (2) history of alopecia or medical conditions with hair loss, except for androgenetic alopecia; (3) known symptomatic cardiac diseases; (4) use of topical finasteride or dutasteride within 6 months; (5) history of allergy to any components listed in the test or the control products; (6) pregnant or breastfeeding women; (7) those who had participated in other clinical trials within 30 days prior to the enrollment; and (8) those with any other medical illness that could interfere with the study. All participants provided written informed consent before the initiation of the study.

**TABLE 1:** The demographic and baseline characteristics.

Characteristics	Test group (*n* = 44)	Placebo group (*n* = 44)	*p* value
Gender			0.8002 (T)
Male (%)	17 (38.64)	17 (38.64)	
Female (%)	27 (61.36)	27 (61.36)	
Age (mean ± SD, years)	38.52 ± 7.98	38.98 ± 8.80	0.8002 (T)
Weight (mean ± SD, kg)	67.21 ± 13.57	66.64 ± 18.34	0.5205 (W)
Height (mean ± SD, cm)	165.43 ± 8.24	165.72 ± 10.00	0.8837 (T)
Duration of hair loss (mean ± SD, years)	8.12 ± 3.38	9.25 ± 2.80	0.1421 (W)

*p* value: two sample *t*‐test (T) or the Wilcoxon rank‐sum test (W).

### 2.2 Study design and test product

The study was designed as a randomized, double‐blinded, placebo‐controlled clinical trial and was performed at the Global Medical Research Center, Seoul, Korea, from September 2021 to April 2021. The study was conducted for 24 weeks and included a total of five visits for each participant (screening, week 0, week 8, week 16, and week 24). The permitted window periods were ±7 days for week 8, 16, and 24.

The active ingredient of the test product, BLH308, was a PLE formulation of green tea and sophora fruit extract, both of which are known for their anti‐inflammatory and anti‐oxidant properties. These extracts were formulated in a 2:1:1 compounding ratio, respectively, considering the contents of tannic acid in PLE (ca. 12.5%), epigallocatechin‐3‐gallate (EGCG, ca. 50%) in green tea extract, and sophoricoside (ca. 3%) in sophora fruit extract. The information regarding the test and placebo products is shown in Table S1.

### 2.3 Measurement of the clinical efficacy of the test product

#### 2.3.1 Hair density and thickness

The efficacy of the test product for improvement in hair density and thickness was evaluated by phototrichogram using Folliscope 5.0 (Lead M, Seoul, Korea) at every visit. The analysis was conducted at a temperature of 20–22°C and a relative humidity (RH) of 45%−55%. The measurement site was selected based on relatively low hair density and was marked with a tattoo, which was a small dot with a diameter of 1 mm. At each subsequent visit, the same area was measured and assessed. Hair density was evaluated by counting the number of hairs within a certain area of 1 cm^2^, and hair thickness was assessed as an average value after measuring five strands of hair around the tattoo.

#### 2.3.2 Hair gloss

Measurement of hair gloss was carried out using Glossymeter GL200 (Courage + Khazaka Electronic GmbH, Köln, Germany), and the surface of new hair was examined using a scanning electron microscope (SEM). On visits two, three, four, and five, five measurements were recorded, and the average values for each visit were evaluated. Then, the hair on the back of the head was collected, and the surface of the new hair was photographed. The photographs taken with SEM were evaluated by three independent investigators based on a 12‐point scale^9^ (Table S2), and the results were evaluated as average values.

### 2.4 Safety assessment

At each visit, subjects underwent physical examinations to assess adverse events caused by the test product. In the clinical trial, adverse events, including abnormalities observed in clinical pathology tests (hematological/blood chemical tests, urine tests), vital signs (blood pressure, pulse), and physical measurements (body weight), were evaluated. Any adverse event during the clinical trial was recorded and assessed in the Case Report Form (CRF). The adverse event occurrence ratio was calculated.

### 2.5 Statistical analysis

The subject data consisted of three types: Safety Set (SS) analysis, Full Analysis Set (FAS) analysis, and Per Protocol Set (PPS) analysis. To evaluate the efficacy of the product, PPS analysis was used as the main analysis method, and FAS analysis was additionally performed. The adverse event ratios associated with the test product were calculated in the SS analysis. The missing data was replaced with the most recent available data according to the last observation carried forward (LOCF) principle. We analyzed the within‐group differences between the baseline characteristics and the efficacy parameters (hair density, thickness, and gloss), which were compared using a paired *t*‐test. Two‐sample *t*‐tests or Wilcoxon rank sum tests were used to evaluate changes in parameters (hair density, thickness, and gloss) according to normality. In addition, a Generalized Linear Model (GLM) was performed, considering exercise as a covariate. A *p* value < 0.05 was considered statistically significant. All statistical analyses were performed using Statistical Analysis System (SAS; version 9.4, SAS Institute Inc., Cary North Carolina, USA).

## 3 RESULTS

### 3.1 Participant information

**FIGURE 1:**
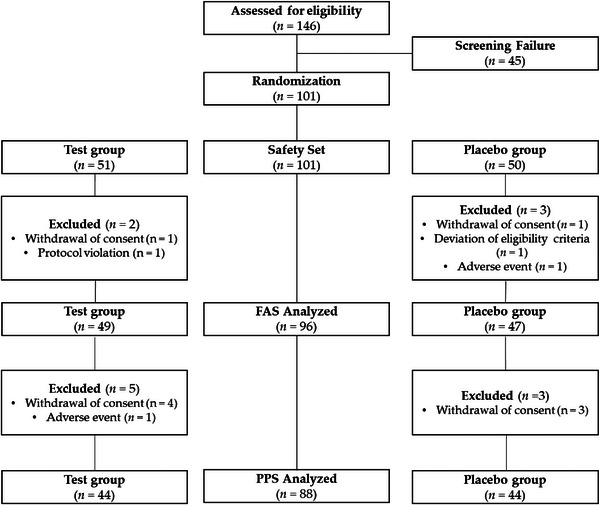
Schematic illustration of the trial protocol.

Figure 1 depicts the study flow with the number of subjects at each stage. A total of 146 subjects were screened initially, and 101 subjects were enrolled and randomly assigned to either the test group (*n* = 51) or placebo group (*n* = 50). Twelve subjects (*n* = 12) were asked to withdraw from the study because they posed a potential risk to subjects, according to investigators’ assessment. The FAS population consisted of 96 subjects (test group: *n* = 49 and placebo group: *n* = 47), and the PPS population consisted of 88 subjects (test group: *n* = 44 and placebo group: *n* = 44). The demographic and baseline characteristics were presented in Table 1.

### 3.2 Clinical efficacy on hair density and thickness

**FIGURE 2:**
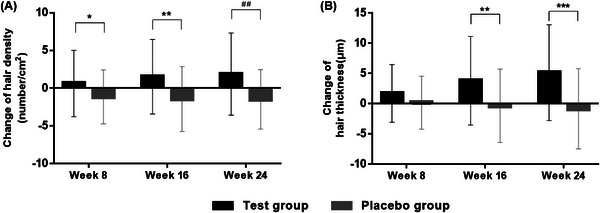
The result of PPS analyzing the change from baseline of hair density (A) and thickness (B) at 8, 16, and 24 weeks after oral consumption of the test product. *p < 0.05, **p < 0.01, ***p < 0.001, Wilcoxon rank sum test; ##*p* < 0.01, paired samples *t*‐test PPS, per protocol set.

During the 24 weeks of oral BLH308 supplementation, we measured hair density at baseline, 8 weeks, 16 weeks, and 24 weeks after taking the test product. In the PPS analysis (Figure 2A), after 8 weeks of intake, the hair density of the test group increased by 0.61 ± 4.39 number/cm^2^ and that of the placebo group decreased by 1.16 ± 3.58 number/cm^2^ compared to baseline. There was a statistically significant difference between the test group and the placebo group (*p* = 0.0201, Wilcoxon rank‐sum test). After 16 weeks of intake, the test group increased by 1.52 ± 4.93 number/cm^2^ and the placebo group decreased by 1.45 ± 4.30 number/cm^2^, indicating significantly different results between the test group and placebo group (*p* = 0.0088, Wilcoxon rank sum‐test). After 24 weeks of intake, the test group increased by 1.86 ± 5.44 number/cm^2^, while the placebo group decreased by 1.48 ± 3.94 number/cm^2^, showing a statistically significant difference between the test group and the placebo group (*p* = 0.0015, paired samples *t*‐test).

In the hair thickness analysis (Figure 2B), after 8 weeks of intake, the test group and the placebo group increased by 1.70 ± 4.77 μm and 0.16 ± 4.38 μm, respectively, but significant differences between the two groups were observed (*p* = 0.0724, Wilcoxon rank‐sum test). Following 16 weeks of intake, the test group increased by 3.80 ± 7.33 μm and the placebo group decreased by 0.36 ± 6.07 μm, showing significantly different results between the test group and the placebo group (*p* = 0.0013, Wilcoxon rank‐sum test). Furthermore, after 24 weeks of intake, the test group increased by 5.14 ± 7.94 μm and the placebo group decreased by 0.86 ± 6.64 μm. indicating a statistically significant difference between the test group and placebo group (*p* = 0.0001, Wilcoxon rank‐sum test).

### 3.3 Clinical efficacy on hair gloss

**FIGURE 3:**
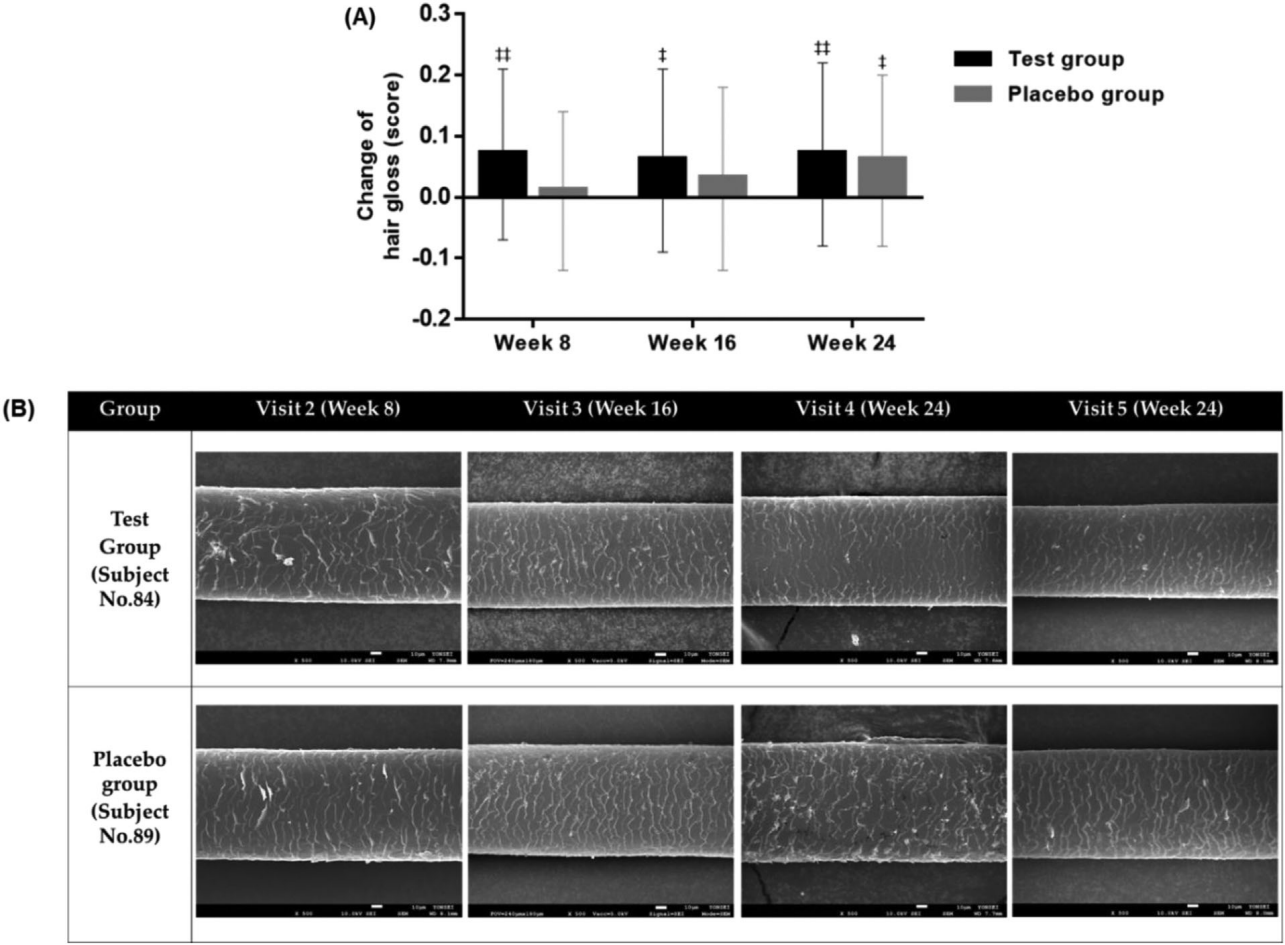
Comparison of change in hair gloss after BLH308 orally ingestion. The result of PPS analyzing the change from baseline of hair gloss at 8, 16, and 24 weeks after oral consumption of the test product. ‡*p* < 0.05, ‡‡*p* < 0.01, Paired *t*‐test (A). Representative images of change in hair surface using SEM. All images were obtained from X500 magnification. PPS, per protocol set; SEM, scanning electron microscope.

In the analysis of the change in hair gloss, we did not find a significant difference in change from the baseline of the test groups compared to the placebo group after 8, 16, and 24 weeks of oral ingestion. However, as shown in Figure 3 we confirmed an increase in the test group when compared to the placebo group. In particular, after 8 weeks of ingestion, the hair gloss was highly improved in the test group compared to the placebo group. The test group significantly increased by 0.07 ± 0.14 scores (*p* = 0.0019, paired *t*‐test), and the placebo group increased by 0.01 ± 0.13 scores (*p* = 0.4742, paired *t*‐test), but no significant differences were observed between the test group and placebo group (*p* = 0.0651, Wilcoxon rank‐sum test).

### 3.4 Adverse events

Safety assessment was conducted by analyzing subjects who had consumed the test product at least once after randomization in a clinical trial. During the clinical trial, there were 31 adverse events in a total of 20 subjects (39.22%) in the test group and 32 adverse events in a total of 22 subjects (44.f00%) in the placebo group during clinical trial, but there was no statistically significant difference between the groups (*p* = 0.6257). Clinical pathological tests were conducted at visits 1 and 5 and were evaluated by dividing them into hematological, blood chemistry, and urine tests (Table S3). No serious adverse reactions occurred, and there were no dropouts due to adverse events.

## 4 DISCUSSION

Persimmon is an East Asian fruit that is renowned for its nutritional value due to its high content of antioxidant compounds, especially flavonoids. Previous studies have confirmed that persimmon leaves contain a wide range of bioactive compounds such as flavonoids, terpenoids, polysaccharides, caffeine, carotenoids, amino acids, vitamin C, minerals, and chlorophylls.^4^ PLE, in particular, is high in phenolic compounds such as proanthocyanidins, quercetin, catechin, and flavonoids, which may contribute to its antioxidant and anti‐inflammatory effects. Since hair growth and aging are influenced not only by genetic and chronological factors, but also by environmental factors such as solar radiation and air pollution, the effects of PLE can result in the long‐term preservation of hair health in general.

In this study, we explored the efficacy and safety of PLE formulated with green tea and Sophora fruit extract (BLH308) on hair growth of healthy adult participants. After 24 weeks of oral intake of the test product, the participants showed significantly increased hair density and thickness compared to the control group. Notably, the hair density showed a significant increase from week 8 compared to the control group, indicating that oral consumption of BLH308 potentially contributed to a relatively early response in hair growth.

The antioxidant and anti‐cancer properties of green tea (*Camellia sinensis*) are well known, and they are mediated by apigallocatechin‐3‐gallate (EGCG), a key component of polyphenols. Previous studies have shown that EGCG potentially contributes to an additive effect on hair growth activity by selectively inhibiting 5α‐reductase activity.^10^ Other investigations have also shown that EGCG promotes hair growth by upregulating phosphorylated Erk and AKT in vivo and in vitro using HFDPCs.^11^ Meanwhile, Sophora fruit (Sophora japonica) possesses estrogenic, anti‐oxidant, anti‐inflammation, and immunomodulatory properties. Its extract contains isoflavone glycosides, which can inhibit the bioactivities of IL‐6 and COX‐2.^12^ These anti‐inflammatory activities might significantly affect the hair growth activity, as shown in previous studies that found increased levels of IL‐6, along with MCP‐3, IFNγ‐inducible protein‐10, and MIP‐1α, in balding zones of androgenetic alopecia compared with non‐balding zones.^13^ Therefore, the formulation of PLE in combination with green tea and Sophora fruit extracts may have additive effects on PLE in term of increasing hair density and thickness.

## 5 CONCLUSION

The oral consumption of PLE formulated with green tea and Sophora fruit extracts showed significantly increased hair density and thickness compared to the control group, showing its possible role in promoting hair growth. Although not statistically significant, the degree of hair gloss in the test group after the oral consumption of BLH308 was also increased compared to that of the control group. As the mechanism involving skin tissue injury caused by extrinsic factors revolves primarily around the installation of oxidative stress, the antioxidative and anti‐inflammatory activities of PLE along with green tea and Sophora fruit extracts could be the major factors resulting in the general improvement of hair health. Further clinical studies with the test product applied to participants with photo‐aged scalps will be necessary to confirm its role in oxidative stress induced by extrinsic aggregations.

## ETHICS STATEMENT

The Institutional Review Board from the study institution approved the study (IRB no. GIRB‐21722‐EA), and informed consents were obtained from each participant before enrolment in the study. This study was registered with the Clinical Research Information Service (CRIS) as PRE20221115‐001.

## Supporting information

Supporting Information

## Data Availability

The data that support the findings of this study are available on request from the corresponding author. The data are not publicly available due to privacy or ethical restrictions.
