# Supplementary material for: Efficacy and safety of persimmon leaf formulated with green tea and sophora fruit extracts (BLH308) on hair growth: A randomized, double‐blind, placebo‐controlled clinical trial
Source: Skin Res Technol. 2023 Aug 22;29(9):e13448. doi: 10.1111/srt.13448 (PMC10443189; doi:10.1111/srt.13448)
Supplement: Supplementary file 1 — Supporting Information [file SRT-29-e13448-s001.docx]

**List of Supplementary Tables**

**Supplementary Table 1**. (A) The content of tannic acid, EGCG, and sophoricoside in BLH308. (B) The information regarding the test and the placebo product.

(A)

|  | **Tannic acid** | **EGCG** | **Sophoricoside** |
| --- | --- | --- | --- |
| **Content (mg/g)** | 66.65 ± 0.33 | 113.96 ± 0.04 | 8.18 ± 0.10 |

(B)

|  | **Raw material** | **mixing ratio (%)** | **Content (mg)** |
| --- | --- | --- | --- |
| **Test product** | BLMo308 | 30.0 | 150.0 |
|  | Maltodextrin | 61.6 | 308.0 |
|  | Silicon dioxide | 1.9 | 9.5 |
|  | Magnesium Stearate | 1.5 | 7.5 |
|  | Gardenia Yellow Color | 5.0 | 25.0 |
|  | **Total** | **100** | **500** |
| **Placebo product** | Microcrystalline Cellulose | 30.0 | 150.0 |
|  | Maltodextrin | 61.6 | 308.0 |
|  | Silicon dioxide | 1.9 | 9.5 |
|  | Magnesium Stearate | 1.5 | 7.5 |
|  | Gardenia Yellow Color | 5.0 | 25.0 |
|  | **Total** | **100** | **500** |

**Supplementary Table S2**. Descriptive criteria of hair surface cuticle condition on a 12-point scale.

| **Grade** | **Criteria** |
| --- | --- |
| **1** | Intact hair |
| **2** | Irregular overlay only |
| **3** | Very gentle lifting up on the edge |
| **4** | Crevices due to visibly lifting up on the edge |
| **5** | Overall lifting up of cuticle layers and slightly crack or hole form |
| **6** | Severe lifting up, crack and hole form |
| **7** | Began to slightly desquamation process of severe lifting up of cuticle layers |
| **8** | Partially visible desquamation form |
| **9** | Overall severe desquamation form |
| **10** | Began to exposure the cortex and rest the one-third of cuticle layers |
| **11** | Partially exposing of the cortex and rest the half of cuticle layers |
| **12** | Exposing of the cortex without cuticle layers |

**Supplementary Table S3.** Hematological and blood chemistry test, before and after the oral ingestion. We analyzed visit 1 with a test group of *n* = 51 and a placebo group of *n* = 50, and visit 5 with a test group of *n* = 44 and a placebo group of *n* = 44.

| **Parameter** | **Group** | **Visit 1 (baseline)** | **Visit 5 (Week 24)** | **Change from baseline** |
| --- | --- | --- | --- | --- |
| **RBC(106/μL)** | **Test group** | 4.57±0.42 | 4.59±0.49 | 0.02±0.22 |
|  | **Placebo group** | 4.58±0.53 | 4.56±0.53 | 0.02±0.22 |
| **WBC(10^3^/μL)** | **Test group** | 5.85±1.64 | 5.84±1.36 | 0.02±1.34 |
|  | **Placebo group** | 5.92±1.68 | 5.63±1.33 | -0.21±1.38 |
| **Hb(g/dL)** | **Test group** | 13.75±1.56 | 13.61±1.62 | -0.18±0.65 |
|  | **Placebo group** | 13.87±1.68 | 13.62±1.77 | -0.17±0.76 |
| **Hct(%)** | **Test group** | 42.84±4.24 | 42.08±4.11 | -0.82±2.15 |
|  | **Placebo group** | 42.81±4.81 | 41.84±4.91 | -0.67±2.42 |
| **Platelet(10^3^/μL)** | **Test group** | 269.57±58.12 | 277.32±62.13 | 12.32±37.60 |
|  | **Placebo group** | 271.36±48.96 | 282.27±55.54 | 9.23±33.34 |
| **Neutrophil(%)** | **Test group** | 56.14±8.88 | 55.90±8.05 | -0.48±9.31 |
|  | **Placebo group** | 55.74±10.17 | 52.36±8.57 | -2.73±10.02 |
| **Lymphocyte(%)** | **Test group** | 34.00±7.97 | 34.22±7.34 | 0.41±8.72 |
|  | **Placebo group** | 34.39±9.72 | 37.17±8.36 | 2.13±8.60 |
| **Monocyte(%)** | **Test group** | 7.09±1.85 | 7.09±1.56 | 0.13±1.50 |
|  | **Placebo group** | 7.31±2.22 | 7.55±2.00 | 0.19±2.04 |
| **Eosinophil(%)** | **Test group** | 2.06±1.88 | 2.10±1.48 | -0.03±1.47 |
|  | **Placebo group** | 1.86±1.37 | 2.22±1.37 | 0.39±1.35 |
| **Basophil(%)** | **Test group** | 0.71±0.32 | 0.69±0.36 | -0.03±0.31 |
|  | **Placebo group** | 0.70±0.39 | 0.70±0.33 | 0.01±0.40 |
| **Glucose(mg/dL)** | **Test group** | 91.90±12.35 | 91.32±9.51 | -1.32±10.89 |
|  | **Placebo group** | 93.18±20.43 | 92.02±15.62 | 1.73±8.87 |
| **Total Protein(g/dL)** | **Test group** | 7.22±0.33 | 7.05±0.31 | -0.18±0.26 |
|  | **Placebo group** | 7.26±0.31 | 7.08±0.32 | -0.17±0.27 |
| **Total Cholesterol(mg/dL)** | **Test group** | 193.65±30.42 | 195.95±36.68 | 1.48±24.19 |
|  | **Placebo group** | 188.64±34.26 | 192.95±31.41 | 6.02±20.49 |
